# Porous ZIF-8@polyacrylonitrile composite beads for iodine capture†

**DOI:** 10.1039/d1ra05223c

**Published:** 2021-09-10

**Authors:** Qiang Yu, Xiaohui Jiang, Zhengjun Cheng, Yunwen Liao, Ming Duan

**Affiliations:** Chemical Synthesis and Pollution Control Key Laboratory of Sichuan Province, China West Normal University Nanchong Sichuan PR China 637009 jxh2314508@163.com 1924163238@qq.com; State Key Laboratory of Oil and Gas Reservoir Geology and Exploitation, Southwest Petroleum University Chengdu Sichuan PR China 610500 mduan@126.com

## Abstract

The safe and effective capture and storage of iodine from nuclear waste is of great significance in industry. This article reports the preparation of a series of millimeter-sized ZIF-8@polyacrylonitrile composite beads with high specific surface area and porosity by the phase inversion method for iodine capture. The composite beads showed a higher capture capacity (4150 mg g^−1^) under excess iodine vapor. The amount of iodine adsorbed in the organic solution is also as high as 643 mg g^−1^, and the adsorption conforms to the Freundlich isotherm and the pseudo-second-order kinetic model. Moreover, composite beads also exhibit higher thermal stability (310 °C). Therefore, ZIF-8@polyacrylonitrile composite beads show great potential as a material for capturing and temporarily storing radioactive iodine.

## Abstract 2

This article reports the preparation of a series of millimeter-sized ZIF-8@polyacrylonitrile composite beads with high specific surface area and porosity by the phase inversion method for iodine capture.

## Graphical abstract

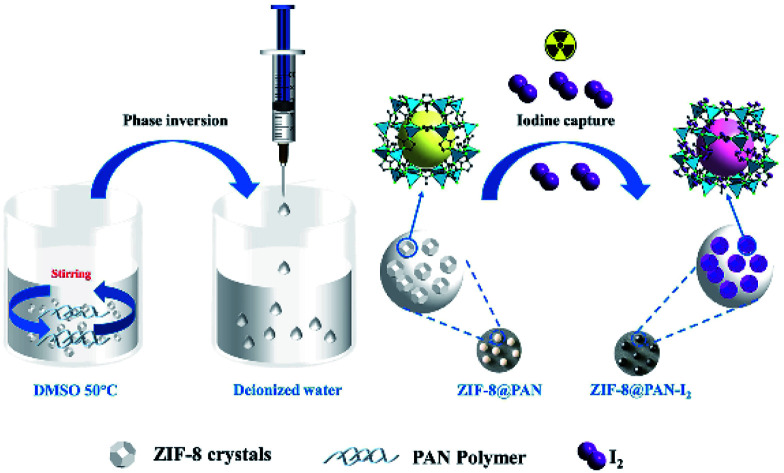

## 1. Introduction

With the development of society and economy, nuclear energy has become one of the most important energy sources, and the safe and effective disposal of nuclear waste has become an important issue.^1^ Radioactive iodine is produced by ^235^U in nuclear fission.^2,3^ Radioactive iodine is widely used in nuclear accident detection, discovery of underground pipeline leaks, medical diagnosis, and treatment, *etc.*^4^ Every coin has two sides. Iodine is easy to accumulate and accumulate in the thyroid.^5^ Once the human body takes in a certain amount of radioactive iodine, it will cause human metabolism disorders and seriously affect human health.^6–9^ Among the radioactive iodine isotopes, the radioactive iodine isotopes that are more harmful to the environment are ^129^I and ^131^I. ^129^I has the characteristics of a high mobility and volatile pollutant with a half-life of 1.52 × 107 years.^10^ It needs to be captured and reliably stored after a long period of decay. In contrast, ^131^I is another highly reactive, volatile and short-lived isotope of iodine with a short half-life (about 8.02 d) and requires immediate capture upon release. Many nuclear leaks (such as the Chernobyl and Fukushima accidents) have shown that the volatile iodine is mainly present in the gas in the form of I_2_.^7,11,12^ Once radioactive iodine leaks out of the nuclear reactor uncontrollably, it will cause serious damage to the local area and even the world.^11^ Therefore, the effective and safe disposal of radioactive iodine has become a topic of great significance.

As far as we know, the adsorption method has been recognized as one of the most effective methods among the methods of capturing iodine. Currently, a variety of techniques have been developed to capture gaseous radioactive iodine from plant exhaust, including wet scrubbing and solid adsorption.^3^ Among the iodine capture methods in waste gas, solid adsorption method has the advantages of simple, reliable, non-corrosive, simple design, low maintenance and operation cost; wet cleaning requires the use of corrosive acids or bases, so the solid adsorption method has advantages over wet cleaning.^3,13^ Nowadays, many materials for capturing iodine have been developed, such as silver-based zeolite,^14–16^ mordenite,^17–20^ activated carbon (AC),^21–23^ porous organic polymer (POPs),^24–26^ metal–organic framework (MOFs),^27–34^*etc.* Currently, among these methods of capturing iodine, the mainstream method is to use silver-based zeolite to capture iodine.^18^ However, due to the high cost, low iodine capture and toxicity characteristics of silver-based materials, their large-scale application in practice is restricted. Metal–organic frameworks (MOFs) are crystalline materials with 1D, 2D or 3D structures,^35,36^ which are formed by the self-assembly of organic ligands and metal ions or clusters through coordination bonds.^37–40^ Recently, a variety of metal–organic frameworks (MOFs) with high specific surface area and porous structure have been proven to be useful for iodine capture.^33,41–44^ Zeolitic Imidazolate Framework-8 (ZIF-8) is a kind of porous crystal material with large specific surface area and high porosity formed by self-assembly of zinc ion and 2-methylimidazole coordination, which ensures good iodine vapor capture ability.^29,45–48^ ZIF-8 also has higher thermal and chemical stability, and its structure is not easily damaged by external influences.^48,49^ In addition, ZIF-8 is relatively low-cost and easy to manufacture, which is helpful for industrial use. However, powdered ZIF-8 is inconvenient to store and transport, and it is difficult to recycle after use, which limits its industrial application. MOFs-based composite beads or composite membranes can solve these limitations.^31,44,50^ Kyriakos C. Stylianou *et al.* reported the use of millimeter-sized HKUST-@polymer beads for iodine capture.^27^ Wen Zhang *et al.* studied MOF-808@PVDF composite beads to capture iodine in gas.^41^ Juan-Tao Jiang *et al.* reported the removal of iodine in solution by PVDF/ZIF-8 nanocomposite membrane,^28^*etc.*

Based on the above work inspiration, coupled with the excellent performance of ZIF-8, we believe that ZIF-8 may be one of the good options for capturing iodine. Therefore, in this work, we prepared ZIF-8 with methanol as a solvent at room temperature, and used polyacrylonitrile (PAN) as a substrate according to our previous work,^18^ and combined them to prepare ZIF-8@PAN composite beads. ZIF-8@PAN composite beads overcome the disadvantages of the powder mentioned above, which makes ZIF-8 more mechanically stable during the I_2_ adsorption process. We conducted a systematic study on the performance of ZIF-8@PAN composite beads to capture iodine, and proposed the mechanism of iodine capture. Our work strongly proves the feasibility of ZIF-8@PAN composite beads to capture iodine, which not only improves the ability of pure ZIF-8 to capture iodine vapor, but is also easy to handle and store, thereby overcoming the disadvantages of powder materials.^27^ Therefore, ZIF-8@PAN material is one of the attractive composite materials that effectively capture iodine.

## 2. Experimental section

### 2.1. Materials

2-Methylimidazole, methanol, iodine (I_2_), polyvinylpyrrolidone (PVP, K30), polyacrylonitrile (PAN, average *M*_w_ 15 000), dimethyl sulfoxide (DMSO) and cyclohexane were purchased from Shanghai Macklin Biochemical Technology Co., Ltd. Zinc acetate dihydrate ((CH_3_COO)_2_Zn·2H_2_O) was purchased from Tianjin Fuchen Chemical Reagent Factory.

### 2.2. Preparation of ZIF-8 crystal

All purchased medicines do not require further purification and are used as they are. ZIF-8 crystals were synthesized according to the literature.^51,52^ In a typical synthesis, first dissolve zinc acetate dihydrate (2.093 g) in methanol (200 mL); then dissolve 2-methylimidazole in methanol (200 mL); finally mix the two solutions at room temperature stir for 5 minutes, then let stand for 24 hours. After that, the supernatant was discarded and centrifuged to obtain a white product, and then methanol was added to repeat the centrifugal washing 3 times, and finally the white product was dried in an oven at 70 °C for 24 hours. The yield of the prepared ZIF-8 sample was 47% (based on Zn).

### 2.3. Preparation of PAN beads and ZIF-8@PAN composite beads

**Scheme 1:**
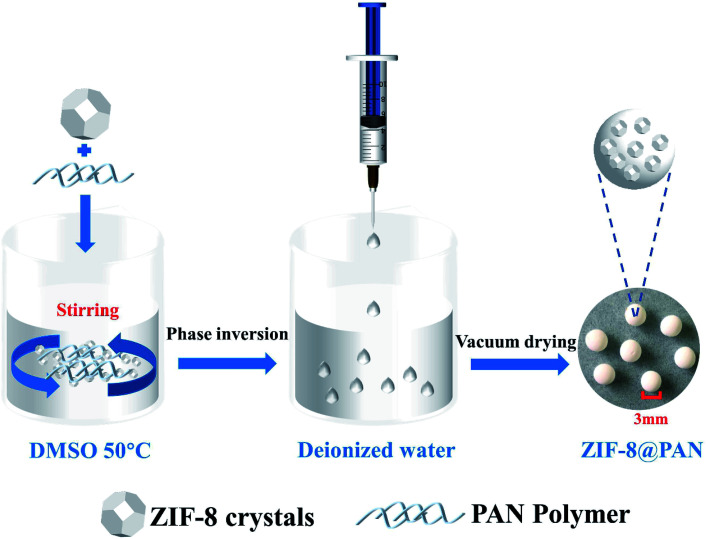
Illustration of the preparation of ZIF-8@PAN composite beads.

ZIF-8@PAN composite beads are prepared according to different ZIF-8 : PAN weight ratios, and PAN beads do not add ZIF-8. The preparation scheme is as follows (Scheme 1): first, dissolve an appropriate amount of polyacrylonitrile (PAN) in an appropriate amount of dimethyl sulfoxide (DMSO) at 50 °C to form a solution. Secondly, add an appropriate amount of surfactant (PVP) to the solution to make PAN fully stirred until it is clear, which is conducive to the full dispersion of the subsequent ZIF-8. Third, add the corresponding amount of ZIF-8 to the above solution and stir, then ultrasound for 30 minutes, and stir again until fully mixed. After that, the mixed solution was dropped from a pipette into deionized water to form solid beads, and after standing for 10 minutes, washing was repeated several times to remove PVP. Finally, the beads were dried in a vacuum oven at 60 °C for 24 hours. A series of composite beads were prepared with seven different ZIF-8 : PAN weight ratios of 1 : 4, 1 : 3, 1 : 2, 1 : 1, 2 : 1, 3 : 1, 4 : 1, respectively labeled as ZIF-8@PAN-1:4, ZIF-8@PAN-1:3, ZIF-8@PAN-1:2, ZIF-8@PAN-1:1, ZIF-8@PAN-2:1, ZIF-8@PAN-3:1, ZIF-8@PAN-4:1. Pure PAN beads are prepared in the same way except that ZIF-8 is not added. By weighing the quality of ZIF-8@PAN composite beads, the measured yields were 96%, 94%, 95%, 94%, 92%, 93%, and 92%, respectively. The specific proportions of the reagents in the preparation process of the composite beads are shown in the following table (Table 1).

**Table 1:** The amount of each reagent used to prepare ZIF-8@PAN composite beads

Samples	ZIF-8 (mg)	PAN (mg)	DMSO (mL)	PVP (mg)
ZIF-8@PAN-1:4	25	100	1.5	6.2
ZIF-8@PAN-1:3	33.3	100	1.5	6.6
ZIF-8@PAN-1:2	50	100	1.5	7.5
ZIF-8@PAN-1:1	100	100	1.5	10
ZIF-8@PAN-2:1	200	100	1.5	15
ZIF-8@PAN-3:1	300	100	1.5	20
ZIF-8@PAN-4:1	400	100	1.5	25
PAN	0	100	1.5	5

### 2.4. Materials characterization

The Fourier transform infrared (FTIR) spectrum of the sample was obtained with a Nicolet-6700 FT-IR spectrometer. Transmission electron microscope (TEM) is used to observe the microstructure of ZIF-8 particles and ZIF-8@PAN samples (FEI Tecanai G2 F20). The scanning electron microscope (SEM) image and energy dispersive X-ray spectroscopy (EDS) of the sample were obtained with a ZEISS GeminiSEM 500 instrument. The PXRD data of the samples were recorded in the range of 3–80° (2*θ*) with a SmartLab X-ray diffractometer. The micromeritics ASAP 2460 instrument was used to record the prepared pore structure and BET surface area at 77 K. Thermogravimetric analysis (TGA) was performed by a thermogravimetric analyzer Labsys evo in a nitrogen atmosphere at a temperature increase/decrease rate of 10 °C min^−1^, and the test temperature range is from room temperature to 800 °C.

### 2.5. Batch adsorption process

#### 2.5.1 Iodine vapor adsorption

The trapping performance of composite beads was proved in iodine vapor and iodine/cycloalkyl solution. In all adsorption experiments, non-radioactive ^127^I was used instead of radioactive iodine, because both have the same electronic configuration and the same potential energy surface, and have similar chemical properties.^53^ All ZIF-8 composite beads were activated at 150 °C before the experiment. According to the following experimental method, the adsorption of iodine vapor under a typical radioactive iodine environment (75 °C, ambient pressure) is simulated: first, put precisely weighed 250 mg of iodine (I_2_) into the bottom of the glass bottle, then put 25 mg of composite beads on the filter paper on the top of the glass bottle, and finally close the lid and seal it. After that, the glass bottle was kept at 75 °C for 8 hours under ambient pressure. After cooling, the composite beads were weighed to estimate the I_2_ adsorption capacity.

The formula for calculating the adsorption capacity of iodine vapor is as follows:1
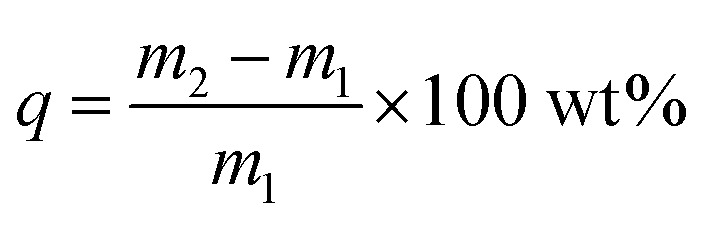
Among them, *q* (wt%) represents the absorption of iodine, and *m*_1_ (mg) and *m*_2_ (mg) represent the mass of the adsorbent before and after the adsorption of iodine vapor.

#### 2.5.2. Adsorption of iodine in liquid phase

The specific experimental process is as follows: first, prepare a series of iodine/cyclohexane solutions of different concentrations. Put a certain amount of ZIF-8@PAN composite beads into a fixed volume of the corresponding concentration of iodine/cyclohexane solution and stir for a certain of time. After the adsorption process is over, the residual concentration of iodine in the supernatant is measured with an ultraviolet-visible spectrophotometer. Finally, calculate the amount of iodine captured by the sample after adsorption equilibrium according to the following formula:2
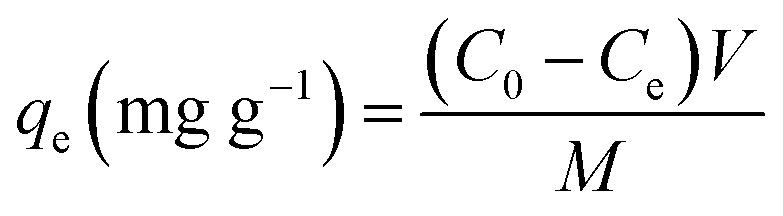
Among them, *C*_0_ (mg L^−1^) and *C*_e_ (mg L^−1^) are the initial iodine concentration and the iodine concentration after adsorption, *V* is the volume of the I_2_ solution (L), and *M* is the mass of the adsorbent (g).

The ZIF-8@PAN composite beads after adsorbing iodine can desorb iodine in ethanol, and the iodine release rate can be calculated by an UV-vis spectrophotometer.

## 3. Results and discussion

### 3.1. Characterization of ZIF-8, PAN and ZIF-8@PAN

**Fig. 1:**
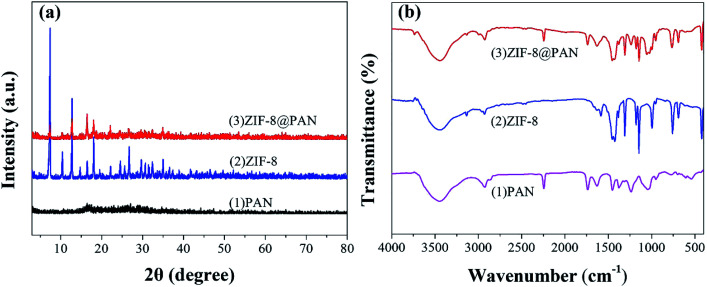
(a) XRD patterns of PAN, ZIF-8and ZIF-8@PAN; (b) FT-IR spectra of PAN, ZIF-8 and ZIF-8@PAN.

The XRD pattern, FT-IR spectrum, SEM image and energy spectrum X-ray energy spectrum (EDS) of the prepared samples were analyzed to confirm the successful preparation of ZIF-8@PAN composite beads. As shown in Fig. 1a, the diffraction peak of the synthesized ZIF-8 crystal sample corresponds to the diffraction peak reported in the literature to confirm the phase purity.^51^ The XRD pattern (Fig. 1a) shows that ZIF-8@PAN composite beads have almost the same characteristic peaks as ZIF-8, but the peak intensity is slightly weaker, which indicates that ZIF-8 crystals have been successfully loaded into PAN beads.^54^ As shown in the FT-IR spectrum (Fig. 1b), the peaks near 2921 cm^−1^ and 2851 cm^−1^ are attributed to the methylene and methyl stretching vibrations in polyacrylonitrile.^55^ The absorption band at about 2245 cm^−1^ is related to the tensile vibration of the –C≡N group in PAN.^55^ The mixed vibration peak of –CN appeared near 1046 cm^−1^.^55^ The absorption peaks near 3335 cm^−1^ and 2921 cm^−1^ are related to the stretching vibration of the methyl group of 2-methylimidazole and the C–H bond in the imidazole ring.^29^ The peak near 1580 cm^−1^ is the tensile vibration of the C=N bond in the imidazole ring,^29^ and the peak near 421.27 cm^−1^ is the tensile vibration of the Zn–N bond.^29^ For ZIF-8@PAN, the peaks in the sample are caused by loading ZIF-8 into PAN. Combined with the FT-IR spectrum analysis of ZIF-8, PAN and ZIF-8@PAN, no new peaks appeared in ZIF-8@PAN. This shows that the process of loading ZIF-8 into PAN to prepare ZIF-8@PAN composite beads only involves physical mixing.

**Fig. 2:**
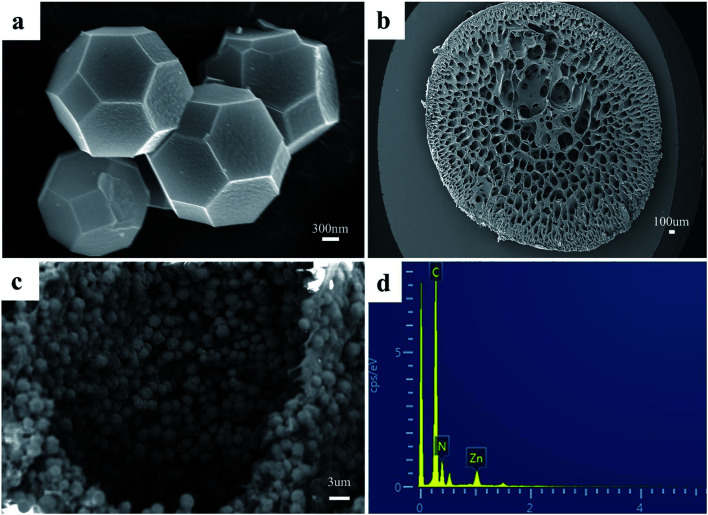
SEM images of (a) ZIF-8, (b) PAN, and (c) ZIF-8@PAN-2:1; (d) EDS of ZIF-8@PAN-2:1.

As shown in Fig. 2a, the synthesized ZIF-8 sample has a dodecahedron structure. According to Fig. 2b, the cross-section of the pure polyacrylonitrile beads reflects the internal hollow porous structure. Fig. 2c shows that the ZIF-8 crystal loaded into the PAN maintains its original form. Fig. S1a1, a2, b1, b2, c1 and c2† show the SEM images of ZIF-8, PAN and ZIF-8@PAN-2:1 at different magnifications. Analysis of the EDS of ZIF-8, PAN and ZIF-8@PAN (Fig. 2d and S2†) showed that Zn is present in the ZIF-8@PAN beads. Combined with Fig. 2, S1 and S2,† it shows that the ZIF-8 crystal has been successfully filled into the inner cavity of the pure PAN beads. In order to fully identify the microstructure of ZIF-8 and ZIF-8@PAN-2:1 samples, HRTEM images were taken through transmission electron microscope (TEM). Fig. S3 and S4† show the HRTEM images of ZIF-8 and ZIF-8@PAN-2:1 samples at different magnifications. It can be inferred that the ZIF-8 particles are wrapped around the PAN, and the ZIF-8 micron-level particles are opaque to electrons. All in all, the above characterization results (XRD, FT-IR, SEM, EDS and HRTEM) proved the successful preparation of ZIF-8@PAN composite beads, and the ZIF-8 in the composite beads retained its original crystal structure.

**Fig. 3:**
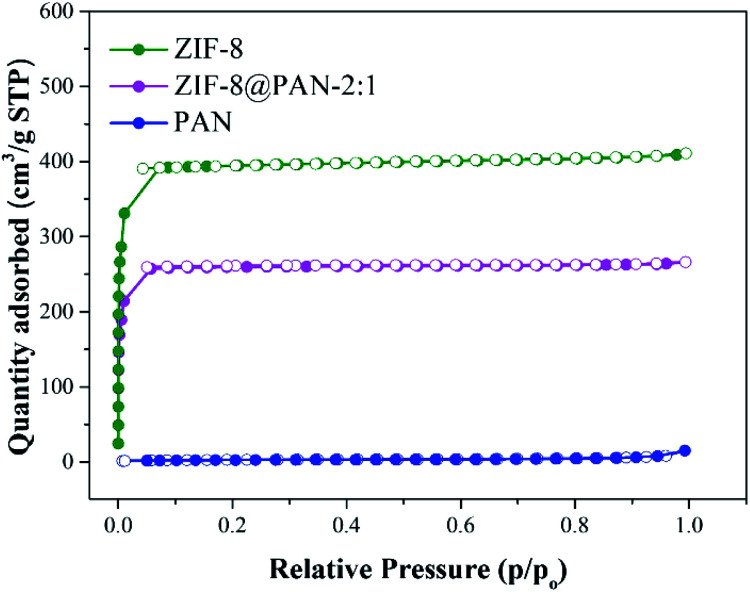
Nitrogen adsorption–desorption isotherms of PAN, ZIF-8@PAN-2:1 and ZIF-8 measured at 77 K.

**Fig. 4:**
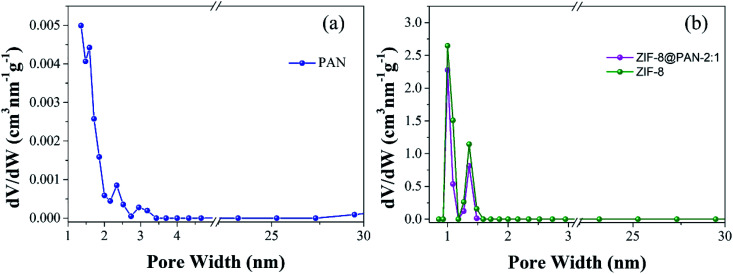
NLDFT pore size distribution of (a) PAN, (b) ZIF-8@PAN-2:1 and ZIF-8.

Brunauer–Emmett–Teller (BET) analysis was used to characterize the porous characteristics of ZIF-8PAN by nitrogen adsorption–desorption method at 77 K. As shown in Fig. 3, ZIF-8 crystals and ZIF-8@PAN-2:1 composite beads exhibit steep nitrogen absorption under low relative pressure (*P*/*P*_0_ = 0–0.01), which indicates that they have a typical type I isotherm. However, PAN beads show obvious adsorption only when the relative pressure (*P*/*P*_0_) is close to 1, showing a typical type III adsorption isotherm. The porosity parameters are shown in Table 2: the BET surface areas of PAN beads, ZIF-8@PAN composite beads and ZIF-8 crystals are 10.0126, 777.4763 and 1193.5031 m^2^ g^−1^, respectively. Compared with ZIF-8 crystals, due to the low loading of ZIF-8 in the polyacrylonitrile carrier, the BET surface area of ZIF-8@PAN-2:1 composite beads is reduced by about 33%. According to the single-point method, the corresponding total pore volumes of the samples were 0.018804, 0.410185, and 0.632431 cm^3^ g^−1^, respectively. As shown in the corresponding NLDFT pore size distribution curve (Fig. 4), (a) PAN pore size distribution is mainly distributed in 1–2 nm, indicating that there is mainly a microporous structure, but also a small amount of mesoporous structure; (b) ZIF-8 and ZIF-8@PAN sample, the pore size is mainly distributed in 1–2 nm, indicating that it is mainly a microporous structure.

**Table 2:** The porous structure parameters of the prepared sample

Materials	*S*_BET_ (m^2^ g^−1^)	*V*_total_ (cm^3^ g^−1^)
PAN	10.0126	0.018804
ZIF-8@PAN-2:1	777.4763	0.410185
ZIF-8	1193.5031	0.632431

### 3.2. ZIF-8@PAN captures iodine in vapor

**Fig. 5:**
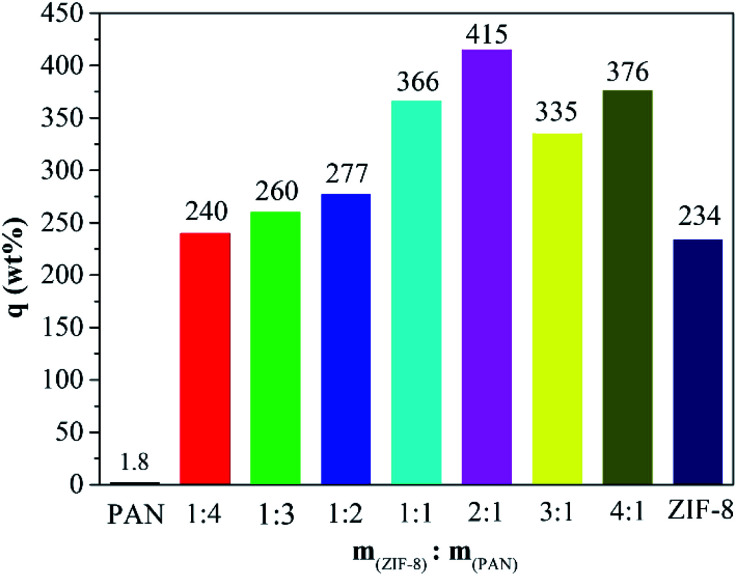
Iodine vapor capture capacity of pure PAN beads, ZIF-8@PAN beads with different ZIF-8 : PAN mass ratios and pure ZIF-8 powder.

As shown in Fig. 5, the maximum captured iodine of the sample beads was calculated according to the gravimetric method. The iodine absorption capacity of pure PAN beads is only 1.8 wt%. However, ZIF-8@PAN-1:4, ZIF-8@PAN-1:3, ZIF-8@PAN-1:2, ZIF-8@PAN-2:1, ZIF-8@PAN-3:1, ZIF-8@PAN-4:1 and ZIF-8 crystals have higher iodine adsorption capacity, 240, 260, 277, 266, 366, 415, 376 and 234 wt% respectively. It seems that as the loading rate of ZIF-8 in the composite beads increases, the ability to capture iodine increases, but when the iodine increases to 3 : 1, the iodine absorption capacity decreases slightly. The composite bead sample formed by embedding ZIF-8 in PAN has a higher ability to capture iodine gas than pure ZIF-8 crystal, which indicates that the combination of the two has a synergistic effect. The introduction of ZIF-8 crystals into polyacrylonitrile will cause its chain sequence to change, thereby forming free cells, through which iodine can reach the surface of ZIF-8 crystals and be captured.^27^ The composite beads with low ZIF-8 content have less ZIF-8 content, so the iodine capture ability is low, while the content of ZIF-8 crystals is too large, and the porosity of the polyacrylonitrile beads decreases, which leads to a decrease in the ability to capture iodine.^29^ The XRD pattern, FT-IR spectrum, SEM image and energy spectrum X-ray energy spectrum (EDS) analysis of the composite beads after capturing gaseous iodine showed that the skeleton of the ZIF-8 crystal had collapsed. Therefore, ZIF-8@PAN composite beads can be used as a temporary storage material for capturing gaseous iodine.

**Fig. 6:**
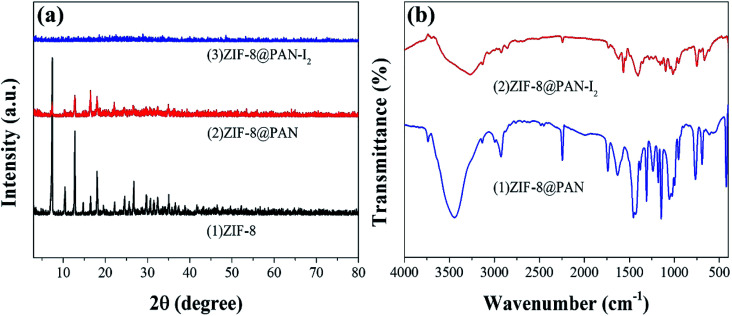
(a) XRD patterns of ZIF-8, ZIF-8@PAN and ZIF-8@PAN–I_2_; (b) FT-IR spectra of ZIF-8@PAN and ZIF-8@PAN–I_2_.

The XRD image of ZIF-8@PAN–I_2_ (Fig. 6a) shows that the characteristic peaks of the ZIF-8 crystal disappeared after iodine absorption, indicating that the crystal structure was destroyed. In the FT-IR image (Fig. 6b) of iodine captured by the composite beads, the characteristic peak near 421.27 cm^−1^ disappeared, indicating that the Zn–N bond was destroyed.

**Fig. 7:**
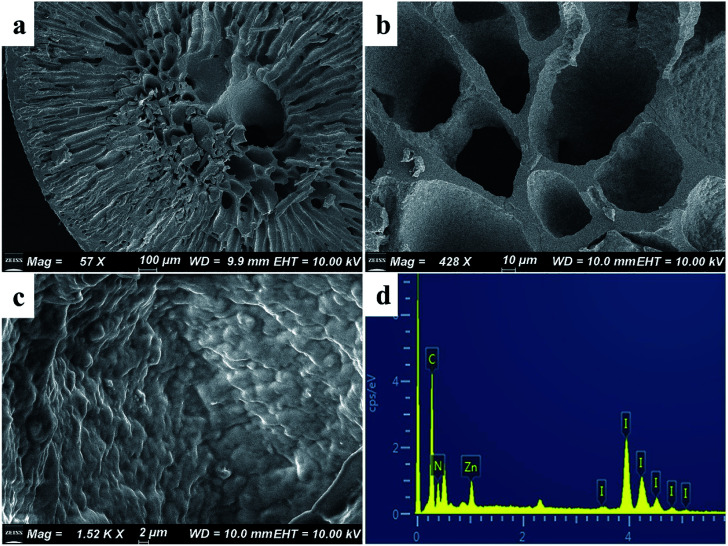
(a–c) SEM images of ZIF-8@PAN–I_2_ at different magnifications; (d) EDS of ZIF-8@PAN–I_2_.

The SEM images of ZIF-8@PAN–I_2_ (Fig. 7a–c) show that the dodecahedral ZIF-8 crystals have disappeared, again proving that the crystal structure of ZIF-8 was destroyed after capturing excess iodine. The EDS of ZIF-8@PAN–I_2_ (Fig. 7d) shows that I_2_ was successfully captured. In summary, after the composite beads capture iodine, the frame of the ZIF-8 crystal in the beads is destroyed after the excessive I_2_ is captured.^29^ Therefore, in practical applications, ZIF-8@PAN composite beads require post-processing after capturing I_2_.^48^

### 3.3. The capture of iodine in organic solution by ZIF-8@PAN

#### 3.3.1 Kinetics study

**Fig. 8:**
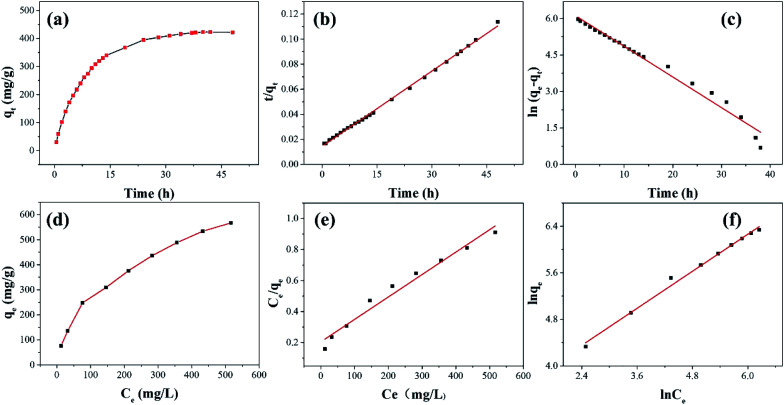
(a) The effect of time on adsorption capacity, (b) pseudo-second-order and (c) pseudo-first-order kinetic model fitting adsorption kinetics (adsorbent: ZIF-8@PAN-2:1, dose = 0.25 g L^−1^, *C*_0_ = 500 mg L^−1^); (d) effect of concentration on adsorption capacity, (e) adsorption isotherms with Langmuir and (f) Freundlich models fitting (adsorbent: ZIF-8@PAN-2:1, dose = 0.25 g L^−1^, time = 48 h).

The non-radioactive I_2_ was dissolved in cyclohexane to prepare an organic solution of iodine for adsorption experiments. The kinetic study of iodine adsorption in solution was carried out in a solution with a concentration of 500 mg L^−1^. The experimental results (Fig. 8a) show that the adsorption capacity increases with time before 24 h, but hardly increases after 24 h, indicating that the adsorption is in equilibrium. During the initial adsorption process, the bead cavity and surface of ZIF-8@PAN-2:1 have enough active sites (ZIF-8), the I_2_ diffusion time is short, and the adsorption capacity increases rapidly; while iodine diffuses into PAN-encapsulated ZIF-8 (Fig. S5†) takes a long time, resulting in slower subsequent adsorption. When the adsorption equilibrium is finally reached, the saturated iodine adsorption capacity of ZIF-8@PAN-2:1 reaches 423 mg g^−1^.

As shown in Fig. 8 and Table 3, the adsorption process of I_2_ by ZIF-8@PAN-2:1 is described by a pseudo-first-order model (eqn (3)) and a pseudo-second-order model (eqn (4)).

3 ln(*q*_e_ − *q*_t_) = ln *q*_e_ − *k*_1_*t*

4
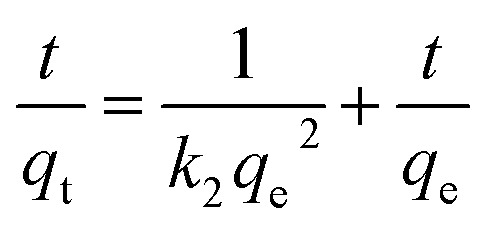
Among them, *q*_t_ and *q*_e_ are the adsorption capacity (mg g^−1^) of the adsorbent at time *t* and equilibrium time I_2_, respectively. *k*_1_ (h^−1^) and *k*_2_ (g mg^−1^ h^−1^) are the adsorption rate constants for the pseudo-first-order and pseudo-second-order kinetic models, respectively.

**Table 3:** The relevant parameters of the pseudo-first-order and pseudo-second-order kinetic models of the iodine adsorption of ZIF-8@PAN-2:1 in I_2_/cyclohexane solution

*q*_e_ (mg g^−1^)	Pseudo-first-order kinetics			Pseudo-second-order kinetics		
423	*k*_1_ (1/h)	*q*_e_ (mg g^−1^)	*R* ^2^	*k*_2_ (g mg^−1^ h^−1^)	*q*_e_ (mg g^−1^)	*R* ^2^
	0.126	453	0.979	2.74 × 10^−4^	500	0.999

The results show (Fig. 8b, c and Table 3) that the correlation coefficient *R*^2^ (*R*^2^ = 0.999) of the pseudo-second-order kinetic equation is relatively large, and the adsorption process of I_2_ on ZIF-8@PAN is best defined as a pseudo-second-order equation.

**Fig. 9:**
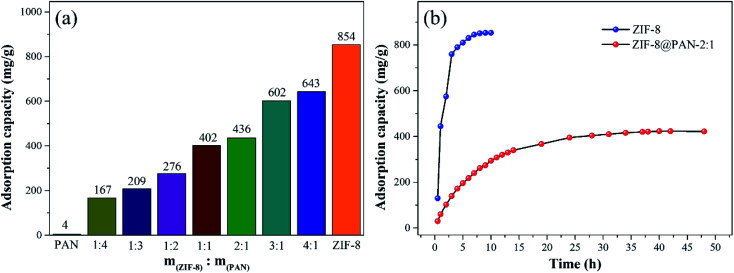
(a) The adsorption capacity of ZIF-8@PAN composite beads with different ZIF-8 content to iodine. (b) Comparison of adsorption capacity and adsorption equilibrium time of ZIF-8 and ZIF-8@PAN composite beads.

**Fig. 10:**
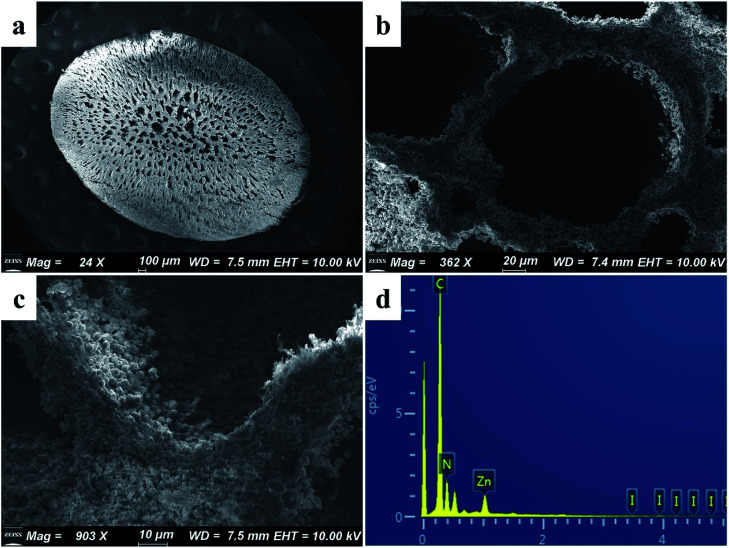
(a–c) SEM images of ZIF-8@PAN-2:1 composite beads at different magnifications after capturing the iodine in the solution; (d) EDS of ZIF-8@PAN-2:1 composite beads after capturing iodine in solution.

**Fig. 11:**
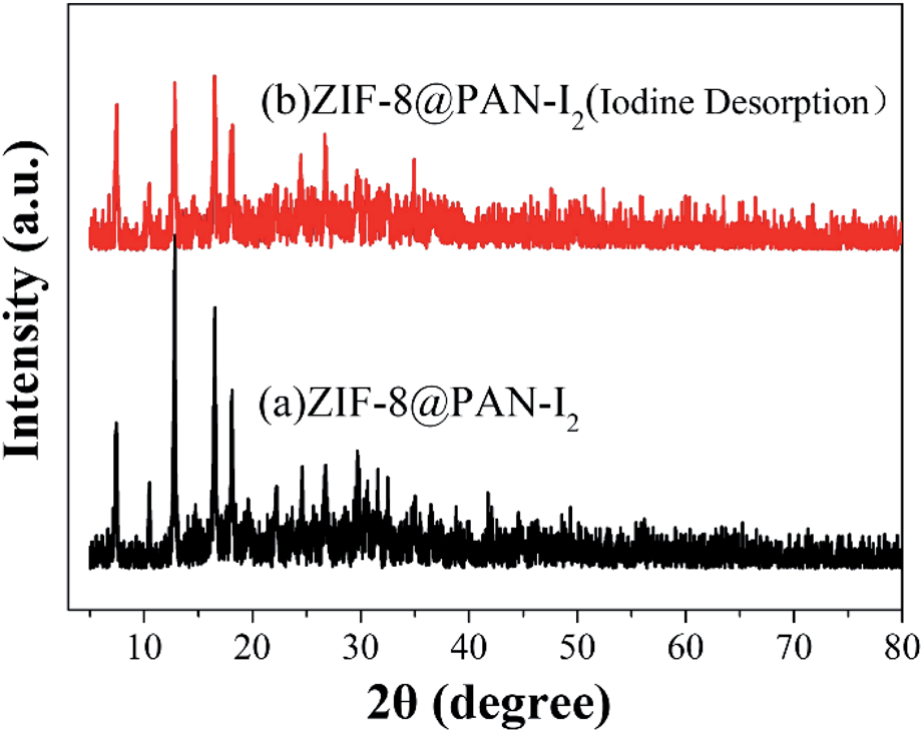
The XRD pattern of ZIF-8@PAN composite beads after absorbing and desorbing iodine in solution.

As shown in Fig. 9a, the iodine adsorption capacity of ZIF-8@PAN composite beads with different ZIF-8 content in a 500 mg L^−1^ I_2_/cyclohexane solution increased with the increase of ZIF-8 loading. The time required for ZIF-8@PAN-2:1 composite beads to reach the equilibrium of iodine adsorption is significantly longer than that of ZIF-8, and the adsorption capacity is lower. This is attributed to the fact that some ZIF-8 in the ZIF-8@PAN composite beads is embedded in the PAN, which causes the I_2_ to diffuse to its surface and be captured for a longer time.^34^ However, since the iodine adsorption capacity of ZIF-8@PAN composite beads is related to the loading of ZIF-8, the larger the load, the greater the adsorption capacity, so the adsorption capacity of composite beads is lower than that of pure ZIF-8 crystals. The ability of ZIF-8@PAN composite beads to capture iodine in I_2_/cyclohexane solution is significantly lower than that of gaseous iodine; due to the low loading of I_2_ (Fig. 5 and 9a), the ZIF-8 inside the composite beads retains the original crystal structure after capturing I_2_ (Fig. 10a–c). As shown in Fig. 10d, the EDS after adsorbing I_2_ from ZIF-8 in the solution has I element present in the composite beads, which indicates the successful capture of I_2_. After iodine absorption, ZIF-8@PAN composite beads can desorb iodine in ethanol, and the iodine recovery rate can reach 69%. The XRD pattern (Fig. 11) shows that ZIF-8@PAN composite beads retain the characteristic peaks of ZIF-8 after absorbing and desorbing iodine in the solution, which proves that it also maintains the original crystal structure.^52^

#### 3.3.2. Adsorption isotherms

As shown in Fig. 8d–f, the adsorption isotherm and isotherm of ZIF-8@PAN-2:1 for iodine are respectively fitted by Langmuir (eqn (5)) and Freundlich (eqn (6)) models.5
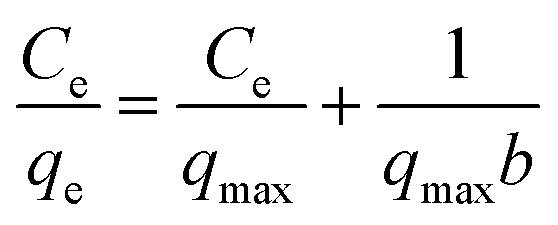

6 ln *q*_e_ = ln *K*_F_ + *n* ln *C*_e_

Among them, *C*_e_ (mg L^−1^) is the concentration of I_2_ in the I_2_/cyclohexane solution at adsorption equilibrium, *q*_e_ (mg g^−1^) and *q*_max_ (mg g^−1^) are respectively the adsorption capacity and saturated adsorption capacity at adsorption equilibrium, *b* (L mg^−1^) is the Langmuir model adsorption coefficient, *K*_F_ is the Freundlich model adsorption coefficient, and *n* is the adsorption intensity coefficient.

According to the isothermal parameters (Table 4) and the fitting line (Fig. 8d–f), the Freundlich model (*R*^2^ = 0.992) is more suitable than the Langmuir model (*R*^2^ = 0.972), indicating that ZIF-8@PAN-2:1 performs multilayer adsorption in this type of adsorption process.^49^

**Table 4:** Langmuir and Freundlich model parameters of I_2_ adsorption on ZIF-8@PAN-2:1

*q*_e_ (mg g^−1^)	Freundlich model			Langmuir model		
436	*n*	*K*_F_ (mg g^−1^)	*R* ^2^	*b* (L mg^−1^)	*q*_max_ (mg g^−1^)	*R* ^2^
	0.530	21.78	0.992	7.06 × 10^−3^	690	0.972

#### 3.3.3. Comparisons of the results by present work and by references

The above experiments show that ZIF-8@PAN composite beads can effectively remove I_2_ in gaseous and solution. It should be compared with other similar materials in order to make effective use of new materials. As shown in Table 5, compared with various iodine adsorbents that have been reported, we can see that ZIF-8@PAN composite beads have good adsorption capacity and faster equilibration time, which are significantly better than many silver-based commercial materials.

**Table 5:** Iodine trapping capacity of various sorbents in this work and references

Adsorbent	Iodine state	*T* (°C)	Eq. time	Adsorption capacity (mg g^−1^)	Ref.
Ag^0^Z	I_2_ (g)	100–200	130–500 h	100–170	14
Ag@Mon-POF	I_2_ (g)	70	—	250	56
Ag-ETS-2	I_2_ (g)	80	72 h	255	57
Cg-5P	I_2_ (g)	140	20 days	870	58
CC3	I_2_ (g)	20	350 h	364	59
SnS_g_	I_2_ (g)	125	25 days	683	1
Al–O–F	I_2_ (g)	90	4 h	10–49	60
MgAl–NO_3_-LDH	I_2_ water solution	RT	2 h	41.2	61
MgO	I_2_ (g)	25	3 h	196	62
KOH–AC	Cyclohexane solution of I_2_	RT	6 h	308	21
ZIF-8	I_2_ (g)	75	12 h	1250	29
ZIF-8@PU	Cyclohexane solution of I_2_	RT	96 h	330	44
HKUST-1@polymer	I_2_ (g)	75	100 h	348–538	27
Cu-BTC@PES	I_2_ (g)	75	75 h	639	34
ZIF-8@PAN	I_2_ (g)	75	38 h	643	This work

### 3.4. Iodine adsorption mechanism

**Fig. 12:**
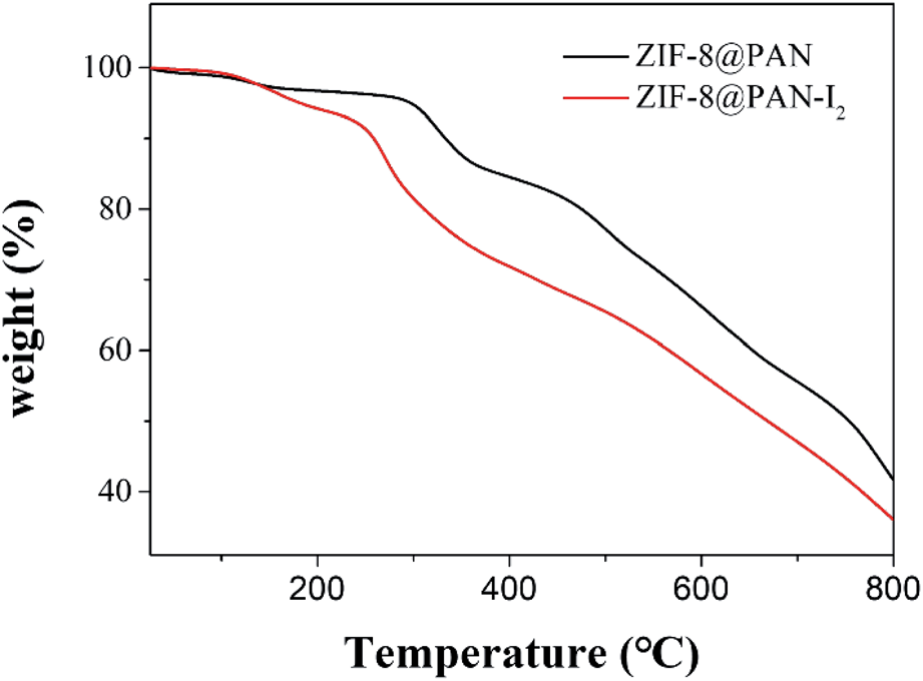
TGA plots of ZIF-8@PAN composite beads and ZIF-8@PAN composite beads after capturing iodine in solution.

Since the capture of iodine by ZIF-8@PAN composite beads as a whole includes PAN and ZIF-8, we will discuss their capture mechanisms separately. First, we will discuss the minute quantity capture of iodine on PAN. According to our previous work, there is only a small amount of physical capture of iodine in PAN.^18^ Through the FT-IR, XRD, EDS, and TG analysis of the ZIF-8@PAN composite beads after capturing I_2_, the mechanism of ZIF-8 capturing iodine was studied, which once again confirmed the previous reports on the mechanism of ZIF-8 capturing I_2_. Dorina F. Sava and his partners^29^ reported in detail the mechanism of ZIF-8 capturing iodine through experiments and molecular simulation calculations. The β cage in the ZIF-8 crystal is connected by a six-membered ring (6 MR) hole with a diameter of 3.4 Å, so the directional diffusion of iodine (3.35 Å) can be achieved, while the smaller four-membered ring (4 MR) hole is too restricted, unable to diffuse any guest molecules. The reaction sites for capturing iodine on the 2-methylimidazole linker of ZIF-8 molecular sieve are the –CH_3_ group and –CH_2_ group on the imidazole ring, through the mutual interaction of I⋯H and I⋯C effect.^29,46,49^ Each ZIF-8 framework can hold up to 5.4 I_2_ molecules. As mentioned above, ZIF-8@PAN composite microspheres capture iodine in an environment with excessive iodine vapor, showing a strong capture ability, but the load of I_2_ exceeds the maximum capacity of the frame, and the ZIF-8 frame will deform or even collapse. However, the amount of iodine captured by ZIF-8@PAN composite beads in I_2_/cyclohexane is lower than the maximum capacity of the frame, and ZIF-8 maintains a good crystal structure (shown in Fig. 10a–c). We performed thermogravimetric analysis on ZIF-8@PAN composite beads and ZIF-8@PAN composite beads after capturing iodine in I_2_/cyclohexane solution. As shown in Fig. 12, the TGA curve of ZIF-8@PAN–I_2_ and the ZIF-8@PAN precursor for comparison. From the TGA curve of ZIF-8@PAN, it can be seen that ZIF-8@PAN has high thermal stability (stable to 310 °C), indicating that it has the prospect of trapping iodine waste gas (150 °C) in practical applications. For ZIF-8@PAN–I_2_, the first drop of approximately 7% observed before 230 °C is due to the removal of iodine physically adsorbed by the composite beads, and the evaporation of adsorbed water and residual solvent in the composite beads. After 230 °C, due to the decomposition of ZIF-8@PAN and the release of I_2_, the mass of the product began to lose a lot, indicating a strong interaction between I_2_ and ZIF-8. It proves once again that the predecessor's conclusion that I_2_ captured before the ZIF-8 crystal frame is not destroyed is firmly bound in the cage.^29,48^ This also shows that ZIF-8@PAN composite beads can be used as a medium for capturing I_2_ and temporary storage.

## 4. Conclusions

In summary, we report a ZIF-8@PAN composite bead with a porous structure that can be used for I_2_ capture in the gas and liquid phases. Through XRD, SEM, TGA and FTIR analysis, we confirmed that ZIF-8 embedded in polyacrylonitrile maintains a good crystal structure. Compared with the easily dispersible ZIF-8 powder, the millimeter-sized ZIF-8@PAN composite beads are easier to store and handle, and are more suitable for practical applications. The thermogravimetric analysis (TGA) results of ZIF-8@PAN composite beads show that it is stable below 310 °C. The ZIF-8@PAN composite beads capture iodine for a long time in an atmosphere with excessive I_2_ vapor, and the loading of I_2_ far exceeds the maximum limit of the ZIF-8 framework, causing the framework to deform or even collapse. While capturing iodine in I_2_/cyclohexane, due to the low I_2_ loading (643 mg g^−1^), the ZIF-8 of the composite beads maintains this good original crystal structure. Our work also provides new ideas for the application of ZIF-8@polymer and even MOF@polymer in the removal of iodine in nuclear waste treatment.

## Conflicts of interest

There are no conflicts to declare.

## Supplementary Material

RA-011-D1RA05223C-s001
