# Supplementary material for: Porous ZIF-8@polyacrylonitrile composite beads for iodine capture
Source: RSC Adv. 2021 Sep 10;11(48):30259–69. doi: 10.1039/d1ra05223c (PMC9041150; doi:10.1039/d1ra05223c)
Supplement: RA-011-D1RA05223C-s001 [file RA-011-D1RA05223C-s001.pdf]

Electronic Supplementary Material (ESI) for RSC Advances.
This journal is © The Royal Society of Chemistry 2021

Porous ZIF-8@polyacrylonitrile Composite Beads for Iodine Capture

Qiang Yu [a,*], Xiaohui Jiang[a,*], Zhengjun Cheng[a], Yunwen Liao[a], Ming Duan[b,*]

[a]Chemical Synthesis and Pollution Control Key Laboratory of Sichuan Province, China West Normal University, Nanchong, Sichuan, PR China 637009.

[b]State Key Laboratory of Oil and Gas Reservoir Geology and Exploitation, Southwest Petroleum University, Chengdu, Sichuan, PR China 610500.

* Corresponding author Email: jxh2314508@163.com (X. Jiang); 1924163238@qq.com (Q. Yu); mduan@126.com (M. Duan).

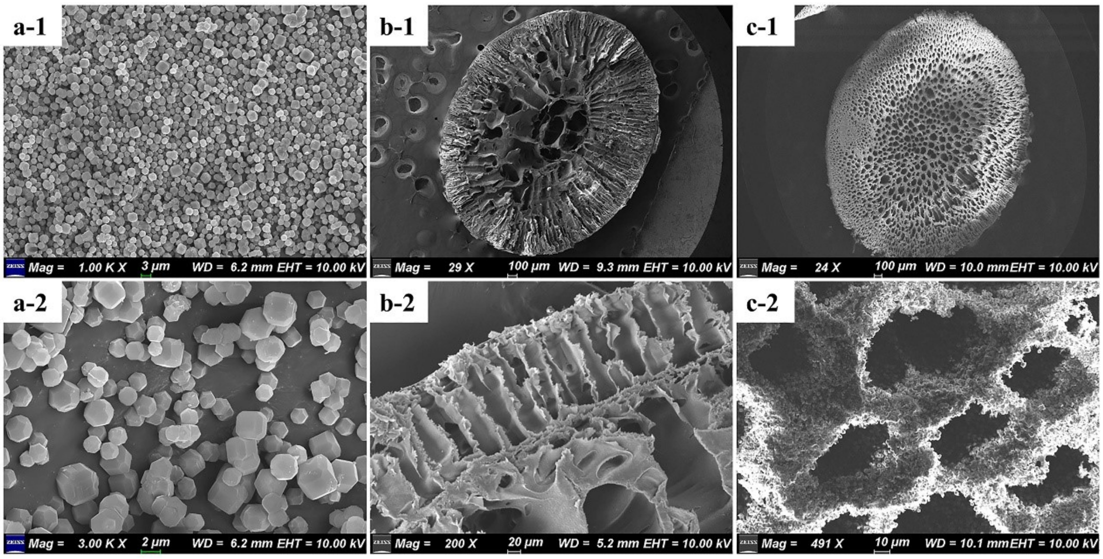

Fig. S1. Figure S1 SEM images of ZIF-8 (a1-a2), PAN (b1-b2), ZIF-8@PAN (c1-c2) at different magnifications.

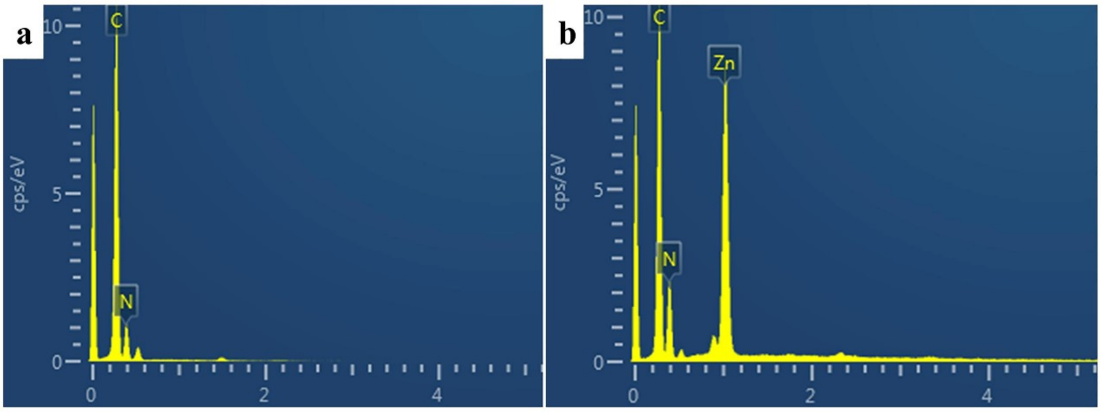

Fig. S2. (a) PAN and (b) EDS of ZIF-8.

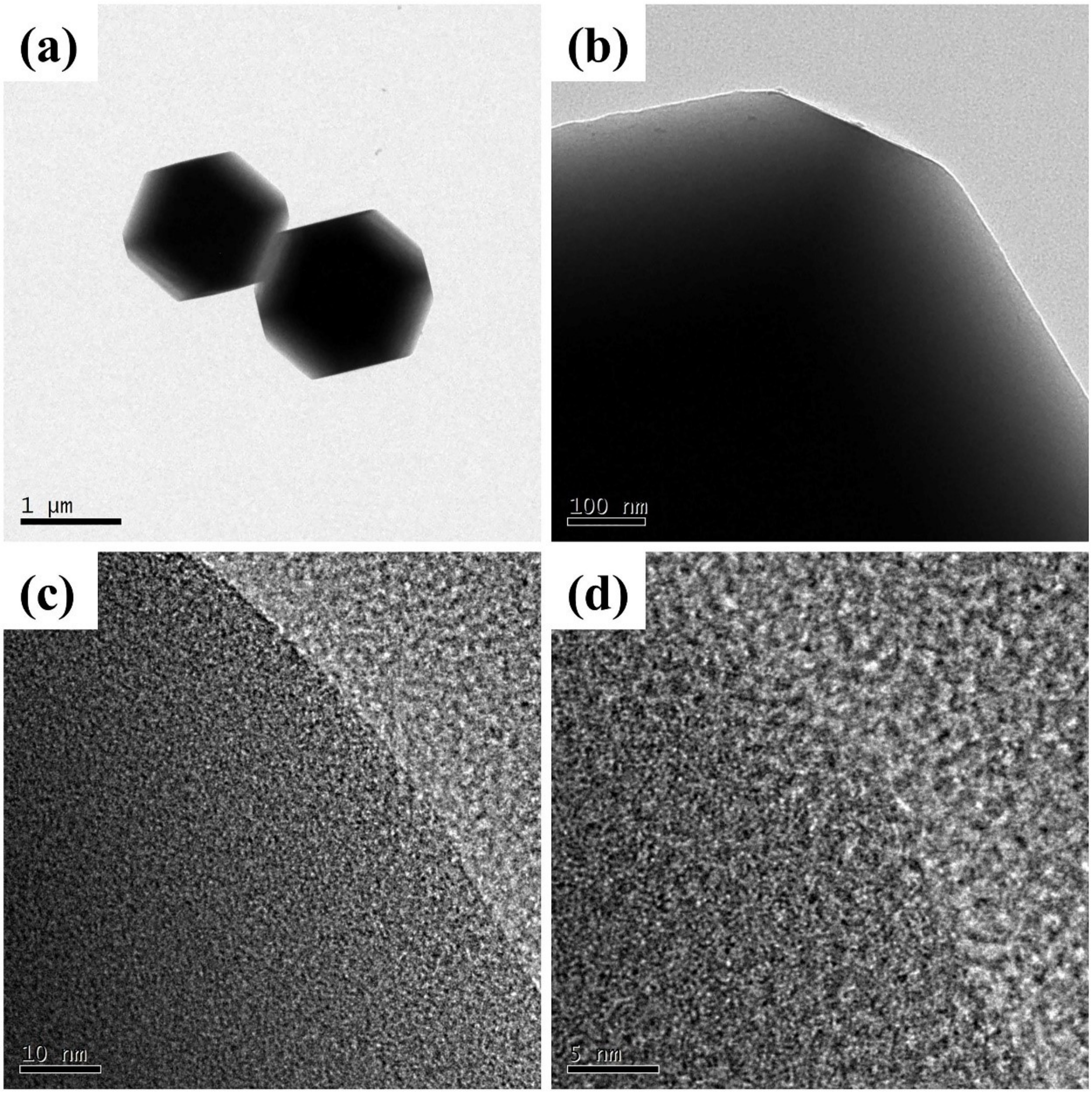

Fig.S3. HRTEM images of ZIF-8 samples at different magnifications.

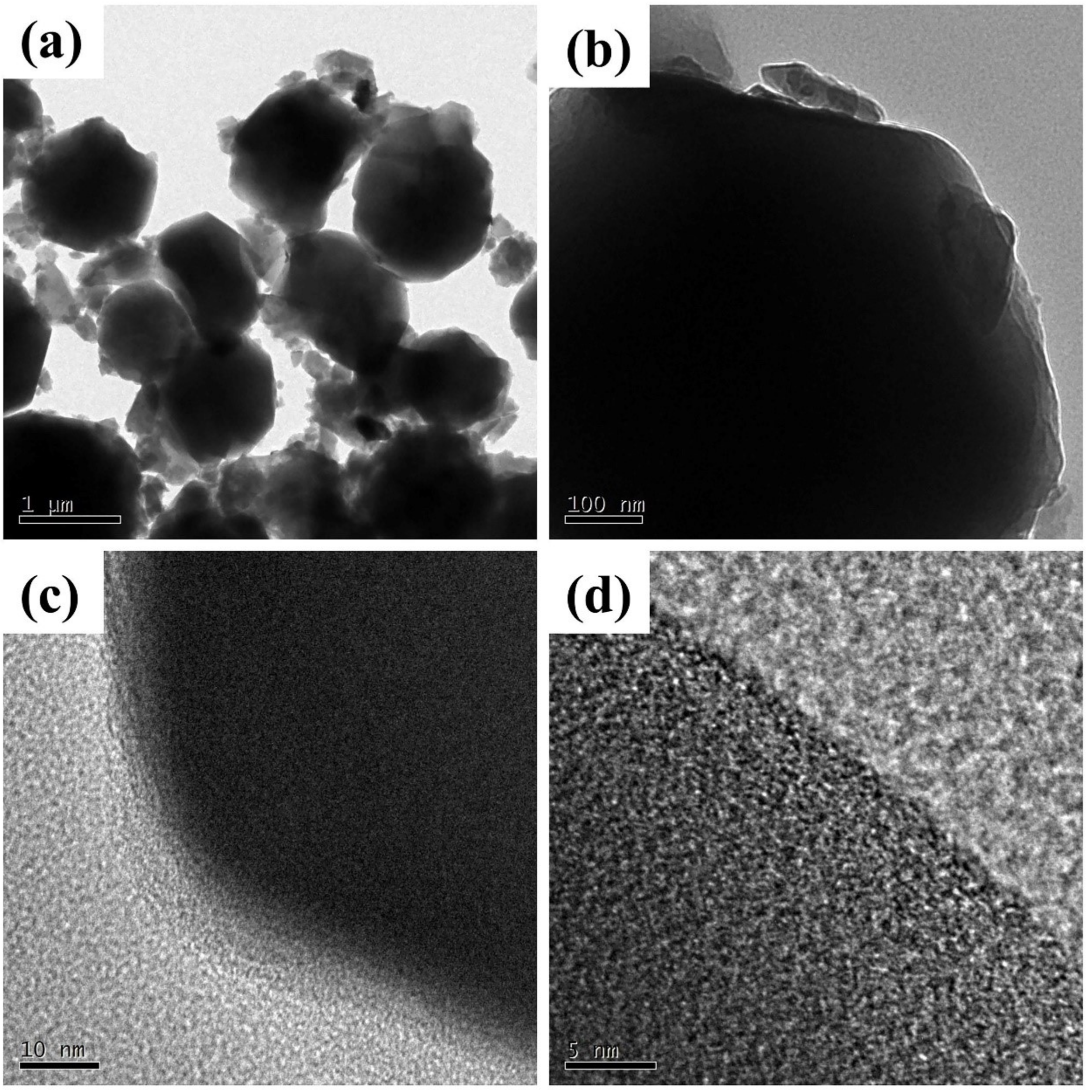

Fig.S4. HRTEM images of ZIF-8@PAN-2:1 samples at different magnifications.

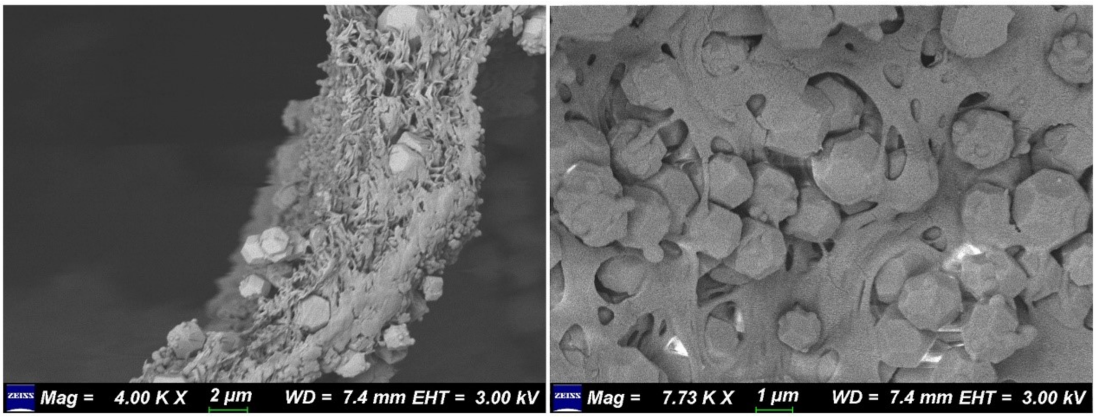

Fig. S5. PAN encapsulated ZIF -8.
